# Stabilized *β*-Catenin Ameliorates ALPS-Like Symptoms of B6/*lpr* Mice

**DOI:** 10.1155/2017/3469108

**Published:** 2017-11-09

**Authors:** Xiaoxie Xu, Jun Huang, Mei Zhao, Huanpeng Chen, Jinhua Mo, Xiaoqing Zhou, Qiao Su, Bolan Yu, Zhaofeng Huang

**Affiliations:** ^1^Institute of Human Virology, Zhongshan School of Medicine, Sun Yat-sen University, Guangzhou, China; ^2^Key Laboratory of Tropical Diseases Control, Ministry of Education in China, Sun Yat-sen University, Guangzhou, China; ^3^Key Laboratory for Major Obstetric Diseases of Guangdong Province, The Third Affiliated Hospital of Guangzhou Medical University, Guangzhou, China; ^4^Animal Experiment Center, The First Affiliated Hospital of Sun Yat-sen University, Guangzhou, China; ^5^Department of Biochemistry, Zhongshan School of Medicine, Sun Yat-sen University, Guangzhou, China

## Abstract

Autoimmune lymphoproliferative syndrome (ALPS) is an incurable disease mainly caused by the defect of Fas-mediated apoptosis and characterized by nonmalignant autoimmune lymphoproliferation. Stabilized *β*-catenin could not only potentiate Fas-mediated T cell apoptosis via upregulating the expression of Fas on activated T cells, but also potentiate T cell apoptosis via intrinsic apoptotic pathway. In the present study, we introduced *β*-cat^Tg^ into *lpr*/*lpr* mice and aimed to explore the potential role of stabilized *β*-catenin (*β*-cat^Tg^) in the development of ALPS-like phenotypes of *lpr*/*lpr* mice. We found that the total splenocyte cells and some compositions were slightly downregulated in *β*-cat^Tg^*lpr*/*lpr* mice, especially the CD4 and CD8 T_EM_ cells were significantly reduced. Meanwhile, stabilized *β*-catenin obviously decreased the numbers of spleen TCR*β*^+^CD4^−^CD8^−^ T (DNT) cells, and the levels of some serum proinflammatory factors also were lowered in *β*-cat^Tg^*lpr*/*lpr* mice. Beyond that, stabilized *β*-catenin slightly lowered the levels of the serum autoantibodies and the scores of kidney histopathology of *β*-cat^Tg^*lpr*/*lpr* mice compared with *lpr*/*lpr* mice. Our study suggested that stabilized *β*-catenin ameliorated some ALPS-like symptoms of *lpr*/*lpr* mice by potentiating Fas-independent signal-mediated T cell apoptosis, which might uncover a potential novel therapeutic direction for ALPS.

## 1. Background

To maintain homeostasis, apoptosis plays a critical role in lymphocyte development and activity. Excessive lymphocyte apoptosis can cause immune defects; however, inhibition of normal apoptosis can lead to the autoimmune disease or the occurrence of lymphoma [1]. So the immune system has developed some apoptotic mechanisms, in which activation-induced cell death (AICD) can be used to inhibit the excessive proliferation of T lymphocytes [1, 2]. Previous study reported that the proteins encoded by Fas and FasL genes were involved in the apoptosis of lymphocytes [3, 4]. As a major suppressing regulation, the AICD controls the homeostasis of T cell during immune response, in which activated T lymphocytes upregulate the expression of Fas (CD95), then interact with the Fas Ligand (FasL, CD95L) on activated B and T lymphocyte surface through the Fas-activating death domain (FADD) to trigger the caspase cascade, leading to proteolysis, DNA degradation, and induction of cell apoptosis [5].

*β*-Catenin, encoded by the CTNNB1 gene, is a multifunctional protein. It has dual roles in the cell, one of which is as the most important information molecule in the canonical Wnt pathway to regulate cell growth, differentiation and apoptosis, and so forth [6]. In the absence of Wnt signal stimulation, the target genes are usually kept inactive and bound by transcriptional corepressors. A cytosolic *β*-catenin “destruction” complex is composed of the adenomatous polyposis coli (APC), axin, and glycogen synthase kinase-3-*β* (GSK3*β*), which marks *β*-catenin for ubiquitination and proteosomal degradation by phosphorylating it in the N-terminus [7]. In response to Wnt signals, binding of Wnt ligands to the frizzled receptors and the lipoprotein receptor-related protein (LRP) coreceptors on the cell surface disrupt the “destruction” complex and inactivation of GSK3*β*-mediated phosphorylation and degradation of *β*-catenin [7, 8]. As a result, nonphosphorylated *β*-catenin is accumulated in the cytoplasm, translocates into the nucleus, displaces the Grg/Tle corepressors, and together with T cell factors leads to the activation of Wnt target genes.

Autoimmune lymphoproliferative syndrome (ALPS) is caused by the defect of Fas-mediated apoptosis and characterized by nonmalignant autoimmunity lymphoproliferation with massive accumulation of lymphocytes and resulting in splenomegaly and lymphoadenopathy [9, 10]. The mutations in the gene encoding Fas (CD95) and Fas-ligand (FasL, CD95L) have been identified in the majority of patients with ALPS [11]. One of the hallmark feature of ALPS is the presence of a large number of TCR*αβ*^+^CD4^−^CD8^−^ double-negative (DN) T cells in peripheral lymphoid organs [12]. *Lpr* and *gld*, two distinct autosomal recessive genes in mice respective with Fas and FasL homozygous mutation, can induce a lymphoproliferative syndrome with the massive accumulation of nonmalignant TCR*αβ*^+^CD4^−^CD8^−^ T cells [4, 13]. The *lpr*/*lpr* and *gld*/*gld* mice have been used as ALPS disease models with the defects of Fas-mediated apoptosis, and they displayed not only a raised DNT subset but also abnormal lymphoproliferation and systemic lupus erythematosus (SLE) [14, 15]. However, the phenotypic expression of the *lpr* and *gld* genes were strongly influenced by background genes [16]. The C57BL/6 background *lpr*/*lpr* mice demonstrated lymphoproliferation, which lacked the expression of Fas on the lymphocyte surfaces caused by a genetic mutation on this gene [17]. There are existing therapeutic approaches for ALPS, most of which use nonspecific immune suppressants to target autoimmune manifestations, but these agents have limitations with long-term administration of the drug and close monitoring for toxicities [18]. It is clear that novel, efficacious therapies are needed for ALPS.

Our previous studies have shown that stabilized *β*-catenin could upregulate the expression of Bcl-xL in CD4^+^CD8^+^ thymocytes to protect them from spontaneous apoptosis and partly upregulate the expression of Fas on activated T cells to promote the apoptosis of them in different mechanisms [19, 20]. Thus, in the present study, we introduced stabilized *β*-catenin into C57BL/6 *lpr*/*lpr* model mice to study the interaction of *β*-catenin-mediated Wnt signaling with Fas-mediated apoptosis in lymphocytes. The results showed that stabilized *β*-catenin ameliorated some ALPS-like symptoms of *lpr*/*lpr* mice, most likely via Fas-independent signal-mediated lymphocytes apoptosis.

## 2. Materials and Methods

### 2.1. Animals

**Figure 1:**
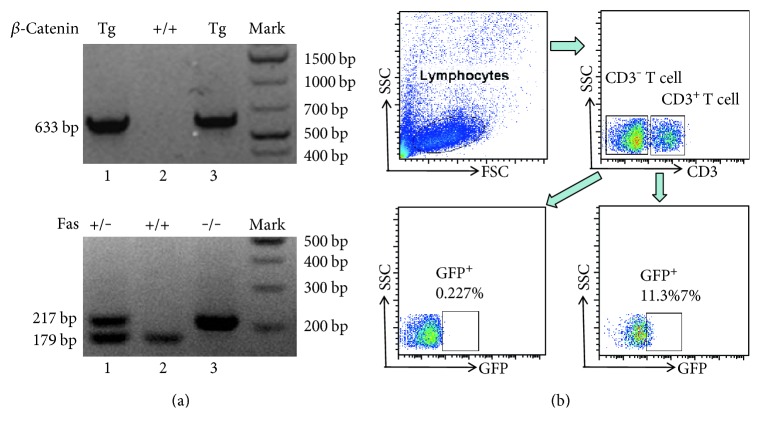
The genotyping results of *β*-cat^Tg^*lpr*/*lpr* mice. When mice was 4 weeks old, the genomic DNA was extracted and used for genotyping. (a) The representative genotyping results of *β*-cat^Tg^ and *lpr*/*lpr* mice. *β*-cat^Tg^ = 633 bp, *β*-cat^WT^ (WT) = 0 bp; Fas mutant (*lpr*/*lpr*) = 217 bp, Fas wild type (WT) = 179 bp, Fas heterozygote (Fas^+/−^) = 217 bp and 179 bp. 1: *β*-cat^Tg^ (*β*-cat^Tg^ FAS^+/−^) mice; 2: WT (*β*-cat^WT^ FAS^+/+^) mice; and 3: *β*-cat^Tg^*lpr*/*lpr* mice. (b) The representative FACS results of *β*-cat^Tg^ mice, respectively, gating on CD3^−^ and CD3^+^ T cell. When the GFP^+^% of T cell is greater than 5%, the mouse is defined as *β*-cat^Tg^ mice.

*β*-cat^Tg^ mice used in this study have been described in previous study [19]. C57BL/6 *lpr*/*lpr* mice were purchased from the National Resource Center for Mutant Mice of China (Nanjing, Jiangsu, China) and crossed with *β*-cat^Tg^ mice to obtain *β*-cat^Tg^*lpr*/*lpr* mice at age 2 months, and the genotypes were identified and displayed in Figure 1. C57BL/6 WT (wild type) mice were purchased from the Laboratory Animal Center of Sun Yat-sen University (Guangzhou, China). In the present study, all used mice were maintained at the specific pathogen-free Animal Research Center under a 12-hour light/12-hour dark cycle at 22°C in Sun Yat-sen University Laboratory Animal Center (Guangzhou, China) and used at age 6 months according to the Institutional Animal Care and Use Committee. All female mice were divided into WT, *lpr*/*lpr*, *β*-cat^Tg^, and *β*-cat^Tg^*lpr*/*lpr* groups, each group with 6 to10 in each experiment.

### 2.2. The Genotype Identification of Offspring Mice

When the pups were 4 weeks, the tail DNA was extracted and used for genotyping. Briefly, 1 cm tail was digested overnight at 55°C in 0.5 ml reagent containing 200 *μ*g/ml proteinase K. Then the DNA samples were extracted according to the conventional protocol, including 500 *μ*l phenol chloroform extraction, 1 : 1 isopropyl alcohol precipitation, 500 *μ*l (75%) ethanol washing, and 100 *μ*l ddH_2_O dissolving. PCR reaction was performed using the Ready Mix Taq solution (Takara, Beijing, China) with an Applied Biosystems (2720 Thermal Cycler, USA). *β*-cat^Tg^ genotyping including *β*-catenin-Tg-K1 and *β*-catenin-Tg-R-K2 two primers, and Fas genotyping including Fas-C, Fas-W, and Fas-K three primers (Table S1 available online at https://doi.org/10.1155/2017/3469108). *β*-cat^Tg^ and Fas genotyping were, respectively, subjected to the following conditions: 994°C 3 min, (94°C 30 s, 60°C 30 s, and 72°C 30 s) for 35 cycles, and 72°C 5 min; 94°C 3 min, (94°C 30 s, 59°C 1 min and 72°C 30 s) for 35 cycles, and 72°C 5 min. The PCR products were separated by electrophoresis using 2% and 3% agarose (BIOWEST®, Chai Wan, Hong Kong) with Gene Ruler™ 1 kb Plus (Fermentas, Shenzhen, China).

### 2.3. Cell Activation and Apoptosis In Vitro

Splenocytes were isolated from 8- to 12-week-old mice. CD4^+^ T cells were purified using a MACS magnetic column with a CD4 T cell enrichment kit (eBioscience, USA). The cells (1 × 10^6^) were cultured for 5 days in fresh medium containing IL-2 (5 ng/ml), and cells (2 × 10^6^) were restimulated in 96-well plates containing different concentrations of plate-bound anti-CD3. Cell cultures were supplemented with IL-2 (5 ng/ml), IL-7(100 ng/ml), and IL-15 (100 ng/ml) to prevent spontaneous apoptosis. Cells were then harvested and washed once with ice-cold PBS supplemented with 1% FCS. Cell pellets were stained with anti-CD3-APC, annexin V, and 7-AAD (BD Biosciences) following the manufacturer's protocol. Analyses were performed on a FACS-caliber (BD Biosciences) with CELLQuest software.

### 2.4. Antibodies and Flow Cytometry

Anti-mouse CD3 (clone PC-61) (QuantoBio), anti-mouse TCR*β* chain (clone H75-597) (eBioscience, San Diego, CA, USA), anti-mouse CD4 (clone RM4-5) (eBioscience), anti-mouse CD8a (clone 53-6.7) (eBioscience), rat anti-mouse CD44 (clone IM7) (BD Biosciences, Franklin Lakes, NJ, USA), and rat anti-mouse CD62L (MEL-14) (BD Biosciences) fluorochrome-labeled antibodies were used for flow cytometry. Single cell suspension of spleen and peripheral blood from the mice was prepared as previously described [21] and stained with the fluorochrome-labeled antibodies, and the fluorescence intensity was analyzed using a FACS Calibur Instrument (BD Biosciences, Franklin Lakes, NJ, USA). FlowJo software version 7.2.5 (TreeStarInt, USA) was used to analyse flow cytometry data.

### 2.5. Serum Cytokines Analysis

To measure the serum cytokine levels of mice, including IL-1*α*, IL-1*β*, IL-2, IL-4, IL-5, IL-6, IL-9, IL-10, IL-12(p40), IL-12(p70), IL-13, IL-17, TNF-*α*, IFN-*γ*, G-CSF, GM-CSF, KC, MCP-1, MIP-1*α*, MIP-1*β*, RANTES, and Eotaxin, we used a Bio-Plex Pro™ Mouse Cytokine 23-Plex panel (Bio-Rad Laboratories, Hercules, CA, USA) according to the manufacturer's protocols. Array analysis was performed using the Bio-Plex® Protein Array system (Bio-Rad Laboratories, Hercules, CA, USA) following manufacturer instructions.

### 2.6. ELISA for Serum Anti-dsDNA Antibody

Blood was collected at the age of 6 months and serum was isolated and stored at −80°C upon use. The levels of serum anti-dsDNA antibody were measured by ELISA with homemade ELISA kit. Shortly, dsDNA-coated plates pretreated with fetal bovine serum blocking (FBS; Hyclone, Logan, UT, USA) were incubated with sample serum in series dilution, then incubated with HRP-conjugated rabbit anti-mouse IgG (whole molecule) secondary antibodies (Sigma, St. Louis, MO, USA). TMB single solution (Life Technologies, Thermo Fisher Scientific, Waltham, MA, USA) was used to develop the color, and the signal was detected on an automatic microplate reader (Thermo Scientific™ Multiskan™ MK3, Waltham, MA, USA) at 450 nm with a reference wavelength of 630 nm.

### 2.7. Histopathology

Kidneys were fixed in 4% paraformaldehyde solution overnight at room temperature and embedded in paraffin. The 4 *μ*m tissue slides were cut and stained with hematoxylin (H&E) or periodic acid schiff (PAS) for routine histological analysis. Sections were visualized by light microscopy at 400x magnification. The H&E and PAS-stained tissue slides were scored blindly by semiquantitative system according to the guidelines previously described [22].

### 2.8. Statistics Analysis

All data are shown as the mean ± SD. The unpaired *t*-test was used to compare two independent groups by the GraphPad Prism software 5.0 (GraphPad Software, San Diego, CA, USA). Significance was achieved when *p* < 0.05.

## 3. Results

### 3.1. Stabilized *β*-Catenin Potentiated the Apoptosis of T Cells of *lpr*/*lpr* Mice

**Figure 2:**
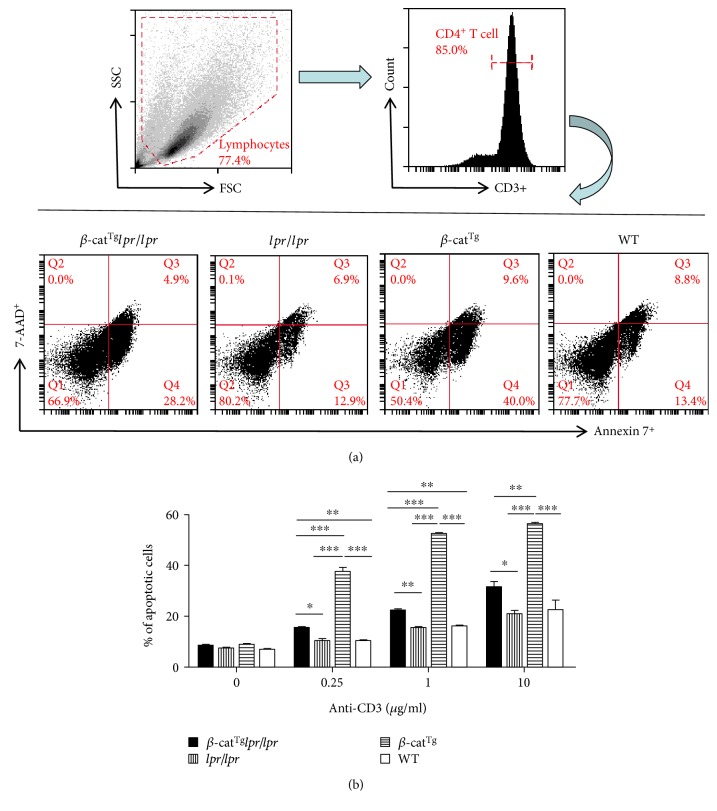
*β*-cat^Tg^ potentiated the apoptosis of T cells of *lpr*/*lpr* mice. WT, *β*-cat^Tg^, *lpr*/*lpr*, and *β*-cat^Tg^*lpr*/*lpr* female mice were sacrificed at age 8–12 weeks and splenocytes were isolated. The CD4^+^ T cells obtained from splenocytes were expanded in IL-2, IL-7, and IL-15 for 5 days. The T cell apoptosis then was induced by using different concentrations of anti-CD3 Ab (*μ*g/ml). (a) The representative FACS results of T cells apoptosis with anti-CD3 Ab (10 *μ*g/ml). The cell pellets were stained with anti-CD3-APC, Annexin V, and 7-AAD surface markers, then the flow cytometry was used to analyze the apoptotic of T cells. (b) The representative statistical analysis of the apoptotic percentages of T cells. Data are shown as mean ± SD (*n* = 3). ^∗^*p* < 0.05; ^∗∗^*p* < 0.01; and ^∗∗∗^*p* < 0.001.

We have found that stabilized *β*-catenin potentiated Fas-mediated T cell apoptosis [20]. To further elucidate the role of transgenic *β*-catenin in the deletion of peripheral T cells, the splenic CD4^+^ T cells of WT, *β*-cat^Tg^, *lpr*, and *β*-cat^Tg^*lpr*/*lpr* mice were challenged with anti-CD3 Ab in vitro. We found that anti-CD3 Ab stimulation significantly increased the apoptosis of CD3^+^ T cells in a concentration-dependent manner (Figure S1). Consistent with previous findings [20], the apoptosis of CD3^+^ T cells were significantly increased in *β*-cat^Tg^ mice compared with WT mice at all concentrations of anti-CD3 Ab stimulation (Figure 2). However, in CD3^+^ T cells from *lpr*/*lpr* mice, the percent of apoptotic cells had no obvious difference compared with WT mice (Figure 2). Beyond that, we found that the apoptotic percent of CD3^+^ T cells was significantly decreased in *β*-cat^Tg^*lpr*/*lpr* mice compared with *β*-cat^Tg^ mice at all concentrations of anti-CD3 Ab stimulation but was higher than that in WT and *lpr*/*lpr* mice (Figure 2). These results demonstrated that *β*-cat^Tg^ could not only potentiate Fas-mediated T cell apoptosis, but also potentiate the apoptosis of T cells in *lpr*/*lpr* mice.

### 3.2. Stabilized *β*-Catenin Slightly Ameliorated the Augments of Splenic Lymphocytes of *lpr*/*lpr* Mice

**Figure 3:**
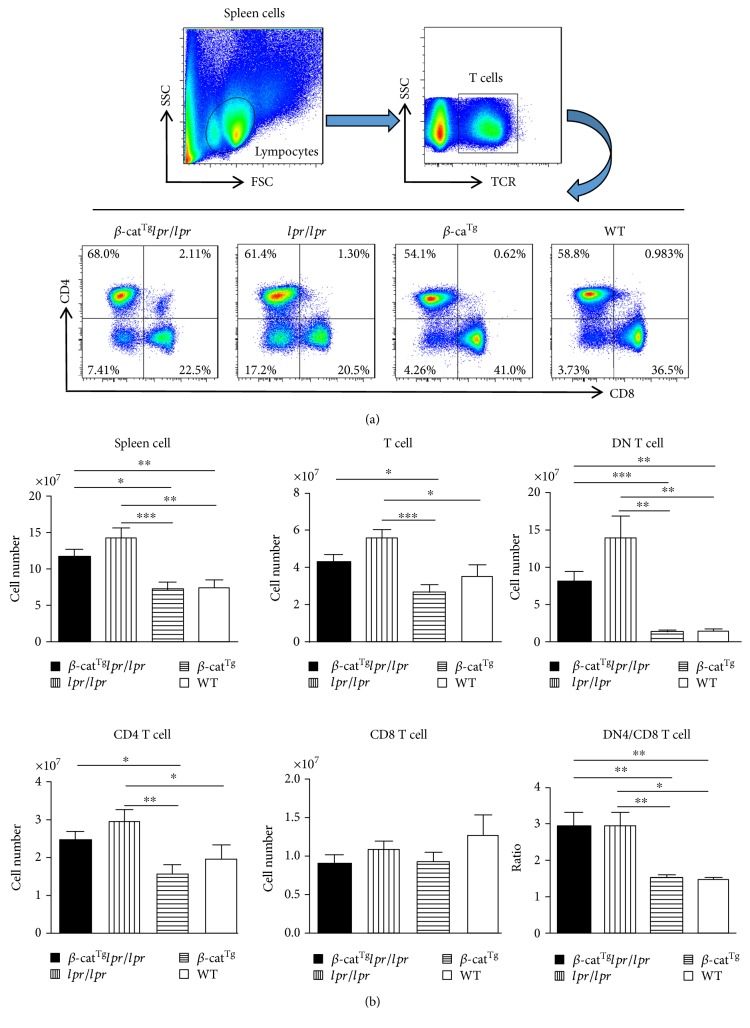
Stabilized *β*-catenin slightly ameliorated the augments of splenic lymphocytes of *lpr*/*lpr* mice. All female animals were sacrificed at age 6 months, and splenocytes were isolated and stained with indicated surface markers. (a) The representative FACS results of CD4^+^ and CD8^+^ T cell subpopulation, gating on TCR*β* positive cell. (b) Statistic analysis of the number of spleen cells, T cells, CD4^+^, CD8^+^, and DNT (TCR*β*^+^CD4^−^CD8^−^) cells and the ratios of CD4 to CD8 in different genotype mice. Data are shown as mean ± SD (*n* = 6–10). ^∗^*p* < 0.05; ^∗∗^*p* < 0.01; and ^∗∗∗^*p* < 0.001.

In the present study, we analyzed the splenic lymphocytes by Flow Cytometry (Figure 3(a)). We found that *β*-cat^Tg^ decreased the numbers of T cells, CD4^+^ T cells, and CD8^+^ T cells compared with WT mice (Figure 3(b)). Meanwhile, we found that the numbers of splenocytes, T cells, and CD4^+^ T cells were significantly increased in *lpr*/*lpr* mice compared with WT mice, but they were slightly declined in *β*-cat^Tg^*lpr*/*lpr* mice compared with *lpr*/*lpr* mice because of stabilized *β*-catenin, although not statistically significant (Figure 3(b)). Moreover, as the remarked change of ALPS, DNT cells were decreased in *β*-cat^Tg^*lpr*/*lpr* mice compared with *lpr*/*lpr* mice, in which they were significantly increased compared with WT mice (Figure 3(b)). These data indicated that stabilized *β*-catenin, at some level, relieved some Fas-mutation-mediated lymphocyte composition changes in *β*-cat^Tg^*lpr*/*lpr* mice.

### 3.3. Stabilized *β*-Catenin Affected the Formation of Memory T Cells

**Figure 4:**
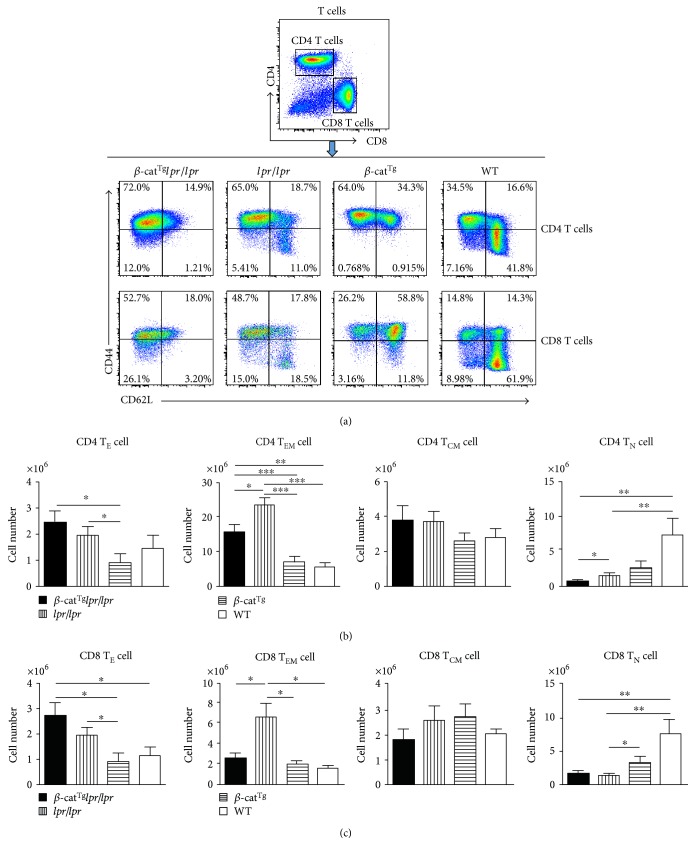
Stabilized *β*-catenin affected the formation of memory T cells. All female animals were sacrificed at age 6 months, and splenocytes were isolated and stained with indicated surface markers. (a) The representative FACS results of memory T cells subpopulation, gating on CD4 and CD8 positive T cell. (b) The numbers of T_E_, T_EM_, T_CM_, and T_N_ cells in CD4 T cells are shown. (c) The numbers of T_E_, T_EM_, T_CM_, and T_N_ cells in CD8 T cells are shown. Data are shown as the mean ± SD (*n* = 6–10). ^∗^*p* < 0.05; ^∗∗^*p* < 0.01; and ^∗∗∗^*p* < 0.001.

We used surface markers CD44 and CD62L to divide spleen CD4^+^ T and CD8^+^ T subpopulations which included effector T cell (T_E_), effector memory T cell (T_EM_), center memory T cell (T_CM_), and naïve T cell (T_N_) (Figure 4(a)). Except for T_CM_, they displayed the same tendency between CD4^+^ and CD8^+^ T cells (Figures 4(b) and 4(c)). In both CD4 and CD8 T cells, the numbers of T_EM_ cells were significantly increased in *lpr*/*lpr* mice compared with WT mice, then rescued by *β*-cat^Tg^ in *β*-cat^Tg^*lpr*/*lpr* mice (Figures 4(b) and 4(c)). However, the T_E_ in *β*-cat^Tg^*lpr*/*lpr* mice was increased compared with *lpr*/*lpr* and WT mice (Figures 4(b) and 4(c)). In addition, either in CD4 or CD8 T cells, the numbers of T_N_ cells in *β*-cat^Tg^*lpr*/*lpr*, *lpr*/*lpr*, and *β*-cat^Tg^ mice were dramatically decreased compared with WT mice, and *β*-cat^Tg^*lpr*/*lpr* mice also displayed a significant decline compared with *lpr*/*lpr* mice in CD4 T_N_ cells, indicating the faster depletion of the subset in *β*-cat^Tg^*lpr*/*lpr* mice (Figures 4(b) and 4(c)). Another long live subset T cell, the T_CM,_ had no significant difference in four groups, either in CD4 or CD8 T cells (Figures 4(b) and 4(c)). These results showed that *β*-cat^Tg^ accelerated the depletion of naïve CD4 T cell and affected the formation of effector memory T cell in different fashions.

### 3.4. Stabilized *β*-Catenin Declined the Levels of Serum Inflammatory Cytokines of *lpr*/*lpr M*ice

**Figure 5:**
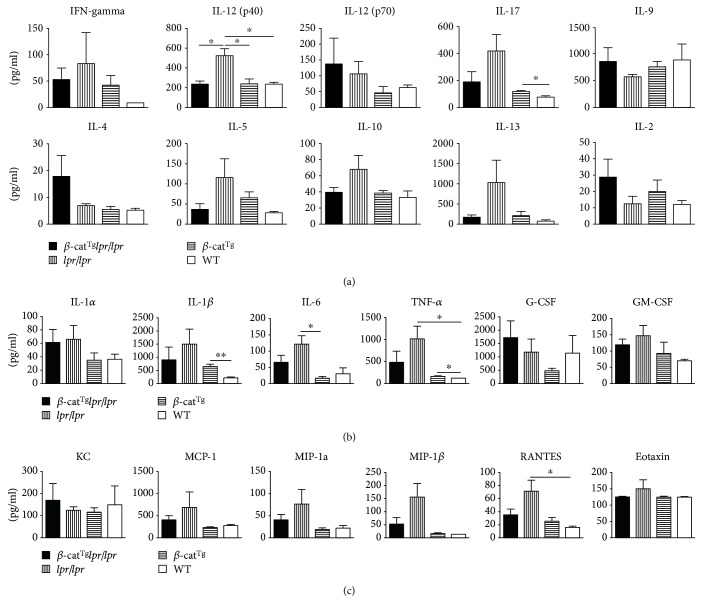
Stabilized *β*-catenin decreased serum inflammatory cytokine level of *lpr*/*lpr* mice. All female animals were sacrificed at age 6 months, and serum were collected, then multiple cytokines level were detected by multiple enzyme-linked immunosorbent assay kit. (a) The levels of comparison of Th1, Th17, Th9, and Th2 cells-related cytokines in serum are shown. (b) The levels of comparison of proinflammatory cytokines and colony-stimulating factors in serum are shown. (c) The levels of comparison of chemokines in serum are shown. Data are shown as mean ± SD (*n* = 3–8). ^∗^*p* < 0.05; ^∗∗^*p* < 0.01.

To analyze the production of cytokine and chemokine in serum, the Bio-Plex Pro Mouse Cytokine 23-Plex panel was used in this study. It is obvious that the concentrations of IFN-*γ*, IL-12(p40), IL-17, IL-5, IL-10, and IL-13 were elevated in *lpr*/*lpr* mice compared with WT mice and rescued by *β*-cat^Tg^ in *β*-cat^Tg^*lpr*/*lpr* mice (Figure 5(a)). In addition, we found that the amount of IL-2 was obviously increased in *β*-cat^Tg^*lpr*/*lpr* mice compared with *lpr*/*lpr* mice (Figure 5(a)). Meanwhile, the inflammatory factors were detected, including IL-1*α*, IL-1*β*, IL-6, TNF-*α*, and GM-CSF, all displayed a lower level in *β*-cat^Tg^*lpr*/*lpr* mice than *lpr*/*lpr* mice, but they were obviously elevated in *lpr*/*lpr* mice compared with WT mice (Figure 5(b)). The inflammatory-related chemokines KC, MCP-1, MIP-1*α*, MIP-1*β*, RANTE, and Eotaxin also were detected; it can be seen that the levels of them, except for KC, were significantly elevated in *lpr*/*lpr* mice compared with WT mice (Figure 5(c)). Whereas, they were obviously declined in *β*-cat^Tg^*lpr*/*lpr* mice compared with *lpr*/*lpr* mice (Figure 5(c)). These results demonstrated that *β*-cat^Tg^ could reduce the levels of serum inflammation-related cytokines and chemokines of *lpr*/*lpr* mice.

### 3.5. Stabilized *β*-Catenin Slightly Ameliorated the Lupus Nephritis of *lpr*/*lpr* Mice

**Figure 6:**
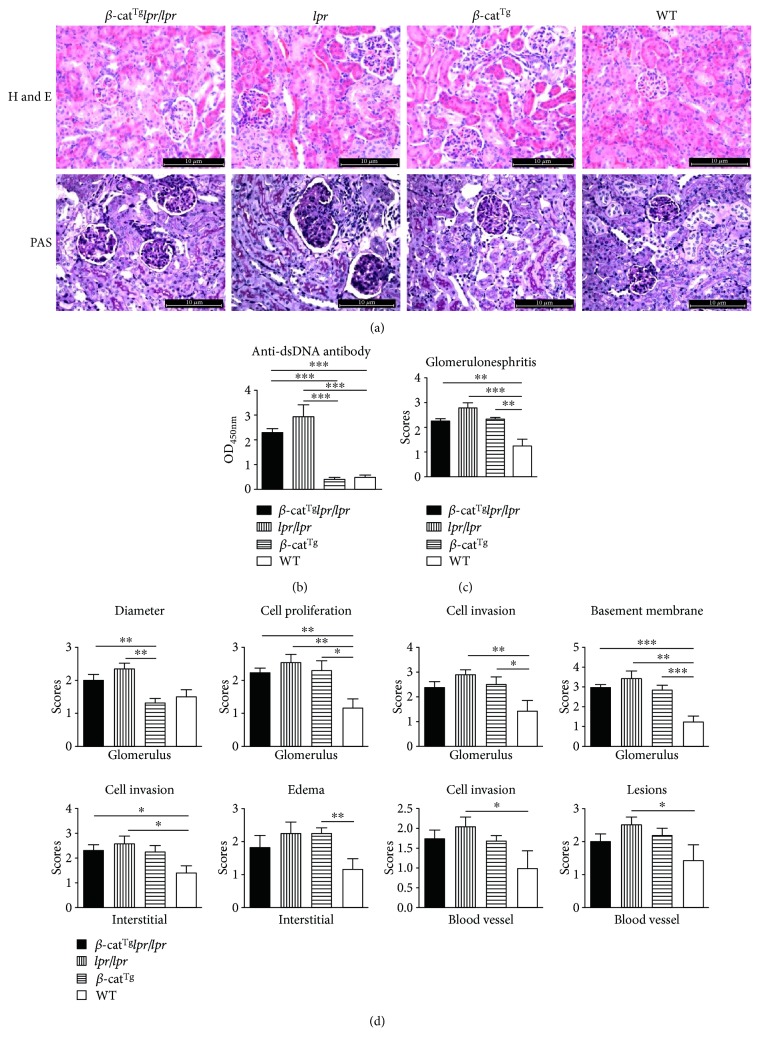
Lupus nephritis was slightly relieved in *β*-cat^Tg^*lpr*/*lpr* mice. All female animals were sacrificed at age 6 months. (a) H&E and PAS staining were used for renal pathology examine. (b) The relative levels comparison of anti-dsDNA antibody in serum. (c) Histopathologic score is shown according to H&E and PAS staining results. (d) The fine grade histopathologic scores for four different groups are shown. Data are shown as mean ± SD (*n* = 6–10). ^∗^*p* < 0.05; ^∗∗^*p* < 0.01; and ^∗∗∗^*p* < 0.001.

As is well-known, lupus nephritis has often been reported in *lpr*/*lpr* mice. The production and accumulation of autoantibodies was one of the important clinical biomarkers of *lpr*/*lpr* mice [23]. Thus, the levels of serum pathogenic anti-dsDNA antibodies were detected in all mice by using the enzyme-linked immunosorbent assay (ELISA). Consistent with previous reports, the level of anti-dsDNA autoantibody was significantly increased in *lpr*/*lpr* mice compared with WT mice (Figure 6(b)). Nevertheless, *β*-cat^Tg^ slightly declined the level of anti-dsDNA autoantibody of *lpr*/*lpr* mice (Figure 6(b)). In the present study, the kidneys of WT, *β*-cat^Tg^, *lpr*/*lpr*, and *β*-cat^Tg^*lpr*/*lpr* mice were processed and stained with H&E and PAS for histomorphologic changes detecting (Figure 6(a)). It is obvious that *β*-cat^Tg^*lpr*/*lpr*, *lpr*/*lpr*, and *β*-cat^Tg^ mice all showed apparently severe kidney damages which were characterized with the mesangial matrix expansion of glomerular, the mesangial matrix increases of glomerular, the inflammatory infiltration of glomerulus, the capillary basement membrane thickening of glomerulus, the crescent formation, the inflammatory infiltration of interstitial, the edema of interstitial, the local sclerosis of vascular, and the inflammatory invasion of perivascular compared with WT mice (Figure 6(a)). The histopathologic scores of *β*-cat^Tg^*lpr*/*lpr*, *lpr*/*lpr*, and *β*-cat^Tg^ mice were significantly higher than WT mice, but *β*-cat^Tg^*lpr*/*lpr* mice had a relatively lower histopathologic score than *lpr*/*lpr* mice (Figure 6(c)). In addition, we performed a fine grade histopathologic score evaluation which include eight subclinical characters and most of them showed the same tendency that *β*-cat^Tg^ slightly declined the scores of *β*-cat^Tg^*lpr*/*lpr* mice (Figure 6(d)). These data implicated that *β*-cat^Tg^ could slightly ameliorate the lupus nephritis of *lpr*/*lpr* mice.

## 4. Discussion

It is well known that postthymic mature T cells express the T cell receptor *α* and *β* chains (TCR*α*/*β*) and also express either CD4 or CD8 surface glycoproteins. Some mature T cells express neither CD4 nor CD8, which were called “double-negative” cells and normally express another form of T cell receptor consisting of *γ* and *δ* chains [24]. However, some studies have identified a very rare subpopulation of T cells which express the TCR*α*/*β* but do not express CD4 or CD8 (TCR*α*/*β* CD4^−^CD8^−^) in *lpr*/*lpr* mice and can induce autoimmune lymphoproliferative syndrome (ALPS) [13]. The accumulation of CD3^+^ T cell receptor (TCR)*αβ*^+^CD4^−^CD8^−^ double-negative T cells (DNT) is a hallmark of autoimmune lymphoproliferative syndrome (ALPS) [25]. Many genetic mutations and the regulator overexpression including Fas, FasL, LPS-responsive and beige-like anchor (LRBA), miR-146a, and IL-12RB1 could result in an ALPS-like phenotype [26–28]. As an incurable disease, ALPS treatments mainly focus on the obliteration of autoimmune disease, lymphadenopathy, lymphoma, and other concurrent diseases. Except for high-dose corticosteroids as common therapy, mammalian target of rapamycin (mTOR) inhibitors sirolimus and pentostatin, Rituximab, and mycophenolate mofetil (MMF) have also been successfully applied in some patients [10]. Many documents have identified ALPS as an example of a genetic disorder of lymphocyte apoptosis caused by mutations in the genes which are related to Fas-mediated cell death pathway, but only few therapeutical strategies for ALPS are relatively known. Indeed, except for the Fas-mediated apoptotic pathway deficiency in ALPS, the role of the intrinsic apoptotic pathway or others in ALPS pathophysiology is not known. *β*-Catenin is a multifunctional protein and is the most important information molecule in the canonical Wnt pathway to regulate cell growth, differentiation and apoptosis, and so forth [6]. Previous studies have shown that *β*-catenin regulates the function of CD8 T cell, and the generation and function of CD8 memory-like T cell also are induced by *β*-catenin transgene [7]. The canonical Wnt/*β*-catenin pathway had been reported to regulate multiple functions, including stem cell regeneration and kidney and reproductive system organogenesis processes [29]. In the present study, we demonstrated that stabilized *β*-catenin slightly rescued some Fas-mutation-mediated ALPS-like manifestations, especially the double-negative T (TCR*β*^+^CD4^−^CD8^−^) cells, by regulating the Fas-independent-mediated apoptosis pathway, uncovering a potential novel therapeutic direction for ALPS.

Previously, we have shown that, in response to staphylococcal enterotoxin B stimulation, Fas crosslinking, or cytokine withdrawal, the T cells from *β*-cat^Tg^ mice were more sensitive to apoptosis compared with WT mice by bounding to Fas promoter to stimulate it and that the levels of Fas on activated T cells were significantly higher in *β*-cat^Tg^ mice than in WT mice, suggesting that stabilized *β*-catenin was able to promote the apoptosis of activated T cells in part by upregulating the expression of Fas on activated T cells [20]. In the present study, we introduced stabilized *β*-catenin into *lpr*/*lpr* mice, to examine the effects of the stabilized *β*-catenin on the survival of peripheral mature T cells in the state of Fas deficiency. We carried out the activation-induced cell death (AICD) experiment by anti-CD3 Ab stimulation in vitro and found that *β*-cat^Tg^ could not only potentiate Fas-mediated T cell apoptosis, but also potentiate the T cell apoptosis in *lpr*/*lpr* mice (Figure 2). We also demonstrated that *β*-cat^Tg^ slightly ameliorated the ALPS-like symptoms of *lpr*/*lpr* mice most likely via Fas-independent signal-mediated apoptosis in vivo. Stabilized *β*-catenin partly reduced the numbers of DNT (TCR*β*^+^CD4^−^CD8^−^) and T_EM_ cells in *lpr*/*lpr* mice (Figures 3 and 4). Especially, *β*-cat^Tg^ obviously reduced the levels of serum inflammation-related cytokines and chemokines in *lpr*/*lpr* mice (Figure 5). Thus, *β*-catenin pathway regulates the survival of peripheral mature T cells by promoting apoptosis in two different mechanisms at least.

Regulated induction of apoptosis is required to maintain T cell homeostasis which is critical for normal functioning of the immune system. In C57BL/6 background *lpr*/*lpr* mice, we found that they showed the ALPS and lupus-like disease symptoms, including progressive accumulation of TCR*β*^+^CD4^−^CD8^−^ T (DNT) cells in spleen, autoantibody production, peripheral mature CD4^+^ and CD8^+^ T cells proliferation, lupus nephritis, and other autoimmune phenomena consistent with previous studies [9, 17, 30]. As a hallmark of the disease, DNT cells can induce autoantibody production, proinflammatory cytokines secretion, and inflamed tissue infiltration [31]. The present of high level autoantibodies to anti-dsDNA also have been linked to the disease progression of SLE [32]. We also found that *lpr*/*lpr* mice had aberrant transcriptional programs and differentiation of CD4^+^ and CD8^+^ T cell subsets, and Fas-negative T cells accumulated not only among DNT, but also among CD4^+^ and CD8^+^ T_EM_ cells, which is consistent with previous studies in ALPS patients [25]. DNT-like cells were detected in both CD4^+^ and CD8^+^ T_EM_ populations and the accumulation of these cells before their double-negative state appears to be an important early event in the pathogenesis of lymphoproliferation in ALPS patients, indicating that DNT can not only derive from CD8^+^, but also from CD4^+^ T cells [25]. Our study also revealed that the numbers of T_N_ cells, either CD4 or CD8, in *β*-cat^Tg^*lpr*/*lpr*, *lpr*/*lpr*, and *β*-cat^Tg^ mice were dramatically decreased compared with WT mice. However, in *β*-cat^Tg^*lpr*/*lpr* mice, the CD4 T_N_ cells displayed faster depletion and the numbers of DNT (TCR*β*^+^CD4^−^CD8^−^) and T_EM_ cells all had a obvious decline compared with *lpr*/*lpr* mice. These results indicated that *β*-cat^Tg^ partly ameliorated the ALPS-like manifestations of *lpr*/*lpr* mice.

In *lpr*/*lpr* mice, SLE also is a manifestation of ALPS. We all know that cytokines play an important role in the pathogenesis of SLE and their balance determines disease activity. For example, the serum IL-6 and IL-10 levels correlate with disease activity [33, 34]. The balance between Th1 cytokines and Th2 cytokines in SLE has been shown to be involved in the pathogenesis of inflammation [35]. In the present study, we found that *lpr*/*lpr* mice had higher IFN-*γ*, IL-12(P40), IL-10, and IL-6 levels in serum than WT mice, which is consistent with previous studies in SLE patients [33]. The levels of IL-17 and IL-13 also had an obvious elevation in *lpr*/*lpr* mice serum compared with WT mice. We also found that proinflammatory cytokine IL-1*β* and TNF*α* were boosted during the process of disease in *lpr*/*lpr* mice serum consisting with previous studies [36]. In addition, the chemokine MIP-1*α* (CXCL3), MIP-1*β* (CXCL4), RANTES (CXCL5), and Eotaxin (CXCL11) also had higher levels. Chemokines are a group of cytokines, whose main action is to recruit leukocyte subsets under homeostatic and pathological conditions. These chemokines play important roles during the inflammation; for example, MIP-17*α* (CXCL3) and RANTES (CXCL5) can bind to receptor CXCR5 and can circulate and migrate immune cells such as neutrophils, monocytes, and effector T cells into inflamed tissues [37]. Over the past decade, the concept of targeting cytokines to treat autoimmune diseases has evolved at a rapid pace, such as some medicines approved by the drug administration to target IL-1*β*, IL-5, IL-6, IL-2, TNF-*α*, and IFN-*γ* [38]. In fact, we found that the levels of IFN-*γ*, IL-12(P40), IL-10, IL-6, IL-17, IL-13, IL-1*β*, TNF*α*, MIP-1*α*, MIP-1*β*, RANTES, Eotaxin, and GM-CSF all had a decline in *β*-cat^Tg^*lpr*/*lpr* mice serum compared with *lpr*/*lpr* mice. Previous study showed that Th1, Th2, and Th17 cells according to the unique functions, respectively, produced IFN-*γ*, IL-4, and IL-17 cytokines [39, 40]. Meanwhile, Th1 and Th17 cells contribute to kidney injury in renal inflammatory diseases [41]. These data suggested that *β*-cat^Tg^ ameliorated the inflammation of *lpr*/*lpr* mice by downregulating multiple inflammatory cytokines/chemokines, and this could be the result of Th1, Th2, and Th17 effects or a combination of effects.

In the present study, the C57BL/6 background *lpr*/*lpr* mice also showed higher concentration of the autoantibody and lupus nephritis, which were the manifestations of SLE [42]. However, *β*-cat^Tg^*lpr*/*lpr* mice demonstrated slightly difference compared with *lpr*/*lpr* mice. In addition, we found that *β*-cat^Tg^ mice demonstrated more severe lupus nephritis, but with a lower concentration of the autoantibody than WT mice. While, previous studies have reported that Wnt/*β*-catenin has a critical role in cell motility and extracellular matrix (ECM) accumulation in tissue fibrosis, and sustained activation of Wnt/*β*-catenin signaling in adult kidney is associated with glomerular and tubulointerstitial epithelial-mesenchymal transition (EMT), leading to the development and progression of renal fibrotic lesions in chronic kidney diseases [43, 44]. At this point, additional studies are required to further identify the exact temporal and spatial effects of stabilized *β*-catenin in renal injury. In summary, *β*-cat^Tg^ and *lpr*/*lpr* mice all showed severe lupus nephritis, but *β*-cat^Tg^*lpr*/*lpr* mice demonstrated relatively slight lupus nephritis (Figure 6). These results demonstrated that *β*-cat^Tg^ ameliorated lupus nephritis of *lpr*/*lpr* mice.

## 5. Conclusions

In summary, we reported herein that stabilized *β*-catenin partly relieved the augments of splenic lymphocytes, the inflammation, and the lupus nephritis of Fas-mutation-mediated ALPS-like manifestations of *lpr*/*lpr* mice, especially suppressed the DNT (TCR*β*^+^CD4^−^CD8^−^) and T_EM_ cells, and declined the levels of some serum inflammation cytokines and chemokines most likely via Fas-independent signal pathway-mediated apoptosis. These findings suggested that stabilized *β*-catenin, in some extent, ameliorated ALPS-like symptoms of *lpr*/*lpr* mice and might uncover a potential novel therapeutic direction for ALPS.

## Supplementary Material

The information of supplementary materials are as follows: Table S1. The sequence of primer for PCR. Fig.S. Apoptosis was induced by anti-CD3 Ab in a dose-dependent manner. All female mice splenic CD4+ T cells were expanded in IL-2, IL-7 and IL-15 for 5 days. The T cells apoptosis then was induced by using different concentrations of anti-CD3 Ab (µg/ml). Cell pellets were stained with anti-CD3-APC, annexin V and 7-AAD surface markers, then used the flow cytometric to analyze the apoptotic of T cells. Data are shown as the mean ± SD(n=3).

## Abbreviations

*β*-cat^Tg^: Stabilized *β*-catenin by genetically modified GFP expression
ALPS: Autoimmune lymphoproliferative syndrome
AICD: Activation-induced cell death
*lpr*: Lymphoproliferation
DNTs: Double-negative T cells
SLE: Systemic lupus erythematosus
ELISA: Enzyme-linked immunosorbent assay
T_E_: Effective T cell
T_EM_: Effective memory T cell
T_CM_: Center memory T cell
T_N_: Naïve T cell
H&E: Hematoxylin
PAS: Periodic acid Schiff
TNF*α*: Tumor necrosis factor *α*
IFN-*γ*: Interferon gamma.
