# Supplementary material for: Stabilized β-Catenin Ameliorates ALPS-Like Symptoms of B6/lpr Mice
Source: J Immunol Res. 2017 Nov 9;2017:3469108. doi: 10.1155/2017/3469108 (PMC5700472; doi:10.1155/2017/3469108)
Supplement: Supplementary file 1 — The information of supplementary materials are as follows: Table S1. The sequence of primer for PCR. Fig.S. Apoptosis was induced by anti-CD3 Ab in a dose-dependent manner. All female mice splenic CD4+ T cells were expanded in IL-2, IL-7 and IL-15 for 5 days. The T cells apoptosis then was induced by using different concentrations of anti-CD3 Ab (µg/ml). Cell pellets were stained with anti-CD3-APC, annexin V and 7-AAD surface markers, then used the flow cytometric to analyze the apoptotic of T cells. Data are shown as the mean ± SD(n=3). [file 3469108.f1.pdf]

**1. Supplementary table**

**Table S1. The sequence of primer for PCR.**

| **Name** | **Primer sequence** |
|---|---|
| **β-catenin-Tg-K1** | 5'-GCCACAAGTTCAGCGTGTCC -3' |
| **β-catenin-Tg-R-K2** | 5'-TCGTCCATGCCGAGAGTGA-3' |
| **FAS-C** | 5'-GFAAATAATTGTGCTTCGTCAG -3' |
| **FAS-K** | 5'-TAGAAAGGTGCACGGGTGTG -3' |
| **FAS-W** | 5'-CAAATCTAGGCATTAACAGTG -3' |

**2. Supplementary figure**

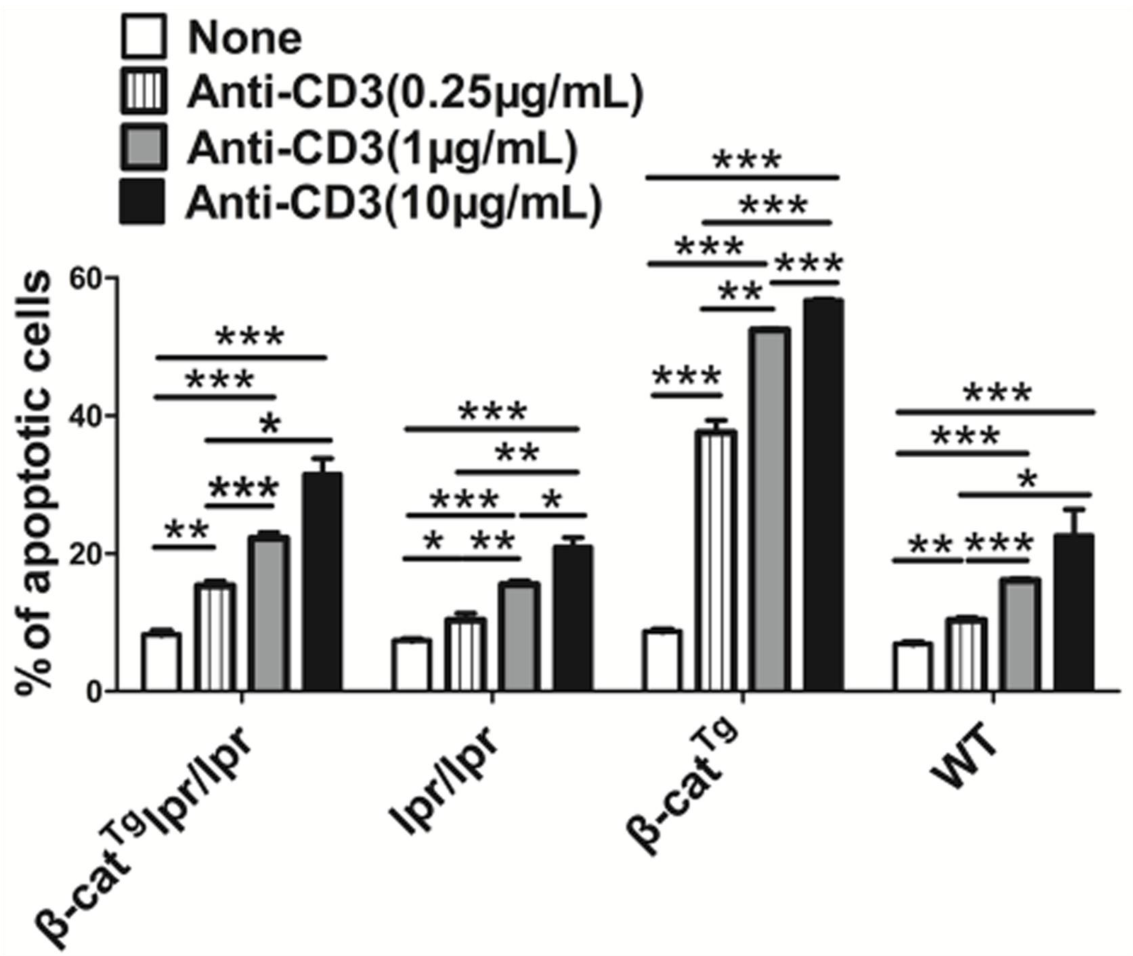


**Fig.S. Apoptosis was induced by anti-CD3 Ab in a dose-dependent manner**. All female mice splenic CD4+ T cells were expanded in IL-2, IL-7 and IL-15 for 5 days. The T cells apoptosis then was induced by using different concentrations of anti-CD3 Ab (μg/ml). Cell pellets were stained with anti-CD3-APC, annexin V and 7-AAD surface markers, then used the flow cytometric to analyze the apoptotic of T cells. Data are shown as the mean ± SD(n=3).
