# Supplementary figures and images for: Stabilized β-Catenin Ameliorates ALPS-Like Symptoms of B6/lpr Mice
Source: J Immunol Res. 2017 Nov 9;2017:3469108. doi: 10.1155/2017/3469108 (PMC5700472; doi:10.1155/2017/3469108)

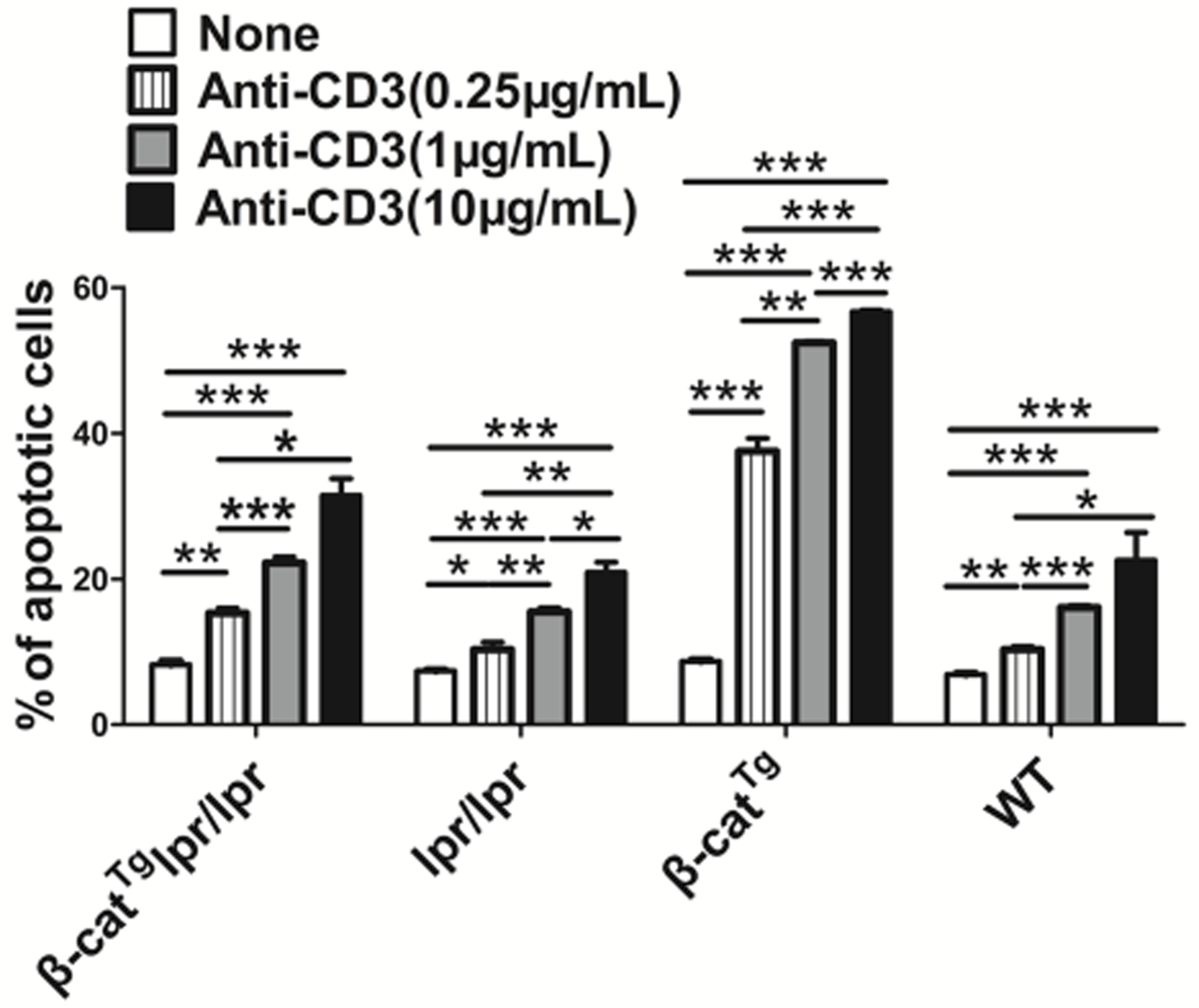

Supplement: Supplementary file 3 [file 3469108.f3.tif]
